# Photo-Responsive Supramolecular Micelles for Controlled Drug Release and Improved Chemotherapy

**DOI:** 10.3390/ijms22010154

**Published:** 2020-12-25

**Authors:** Fasih Bintang Ilhami, Kai-Chen Peng, Yi-Shiuan Chang, Yihalem Abebe Alemayehu, Hsieh-Chih Tsai, Juin-Yih Lai, Yu-Hsuan Chiao, Chen-Yu Kao, Chih-Chia Cheng

**Affiliations:** 1 Graduate Institute of Applied Science and Technology, National Taiwan University of Science and Technology, Taipei 10607, Taiwan; fasihilhami17@gmail.com (F.B.I.); mushroomhaha20@gmail.com (Y.-S.C.); yihalem2000@gmail.com (Y.A.A.); h.c.tsai@mail.ntust.edu.tw (H.-C.T.); jylai@mail.ntust.edu.tw (J.-Y.L.); 2 Graduate Institute of Biomedical Engineering, National Taiwan University of Science and Technology, Taipei 10607, Taiwan; Perry831211@hotmail.com; 3 Advanced Membrane Materials Research Center, National Taiwan University of Science and Technology, Taipei 10607, Taiwan; 4 R&D Center for Membrane Technology, Chung Yuan Christian University, Chungli, Taoyuan 32043, Taiwan; 5 Department of Chemical Engineering, University of Arkansas, Fayetteville, AR 72701, USA; ychiao@uark.edu

**Keywords:** combination chemotherapy, light-controlled drug release, photodynamic therapy, supramolecular micelle, self-assembly

## Abstract

Development of stimuli-responsive supramolecular micelles that enable high levels of well-controlled drug release in cancer cells remains a grand challenge. Here, we encapsulated the antitumor drug doxorubicin (DOX) and pro-photosensitizer 5-aminolevulinic acid (5-ALA) within adenine-functionalized supramolecular micelles (A-PPG), in order to achieve effective drug delivery combined with photo-chemotherapy. The resulting DOX/5-ALA-loaded micelles exhibited excellent light and pH-responsive behavior in aqueous solution and high drug-entrapment stability in serum-rich media. A short duration (1–2 min) of laser irradiation with visible light induced the dissociation of the DOX/5-ALA complexes within the micelles, which disrupted micellular stability and resulted in rapid, immediate release of the physically entrapped drug from the micelles. In addition, in vitro assays of cellular reactive oxygen species generation and cellular internalization confirmed the drug-loaded micelles exhibited significantly enhanced cellular uptake after visible light irradiation, and that the light-triggered disassembly of micellar structures rapidly increased the production of reactive oxygen species within the cells. Importantly, flow cytometric analysis demonstrated that laser irradiation of cancer cells incubated with DOX/5-ALA-loaded A-PPG micelles effectively induced apoptotic cell death via endocytosis. Thus, this newly developed supramolecular system may offer a potential route towards improving the efficacy of synergistic chemotherapeutic approaches for cancer.

## 1. Introduction

Cancer—remains a significant cause of death, with an unprecedented 9.6 million cancer deaths in 2018 [1]. Conventional single-modality cancer treatments are frequently unsatisfactory in many types of cancer due to the complex structure and ability of tumors and the tumor microenvironment to evolve [2,3]. Moreover, the treatment outcomes of chemotherapy are substantially compromised by non-specific product delivery, low bioavailability and poor target precision [4,5]. To resolve these issues, a number of soft materials including lipids, polymers and liposomes have been actively used as drug delivery carriers to improve the blood circulation time and improve the bioavailability of anticancer drugs. These strategies take advantage of the enhanced permeability and retention (EPR) effect, which leads to accumulation of drugs at tumor sites [6]. Several nanomaterials have recently been shown to promote the induction of oxidative stress by upregulating autophagy via the protein kinase B (Akt)/mammalian target of rapamycin (mTOR) pathway [7,8]. Nonetheless, nanoparticles have limited ability to effectively deliver therapeutic agents into tumors, as most nanoparticles become entrapped in the basement membrane of the cells [9,10].

Several research groups have focused on the construction of nano-sized micelles with the potential to accurately identify cancer cells [11,12,13]. Stimuli-responsive polymeric materials (SRPMs) have received a great deal of interest, as their architectures and physical characteristics can be altered by specific stimuli and restored when another stimulus is applied [14,15]. The use of SRPMs for drug delivery has been strongly recommended: smart SRPM-based micelles can react to internal stimuli (pH, enzyme and redox) and external stimuli (temperature, ultrasound and light) within the tumor microenvironment, and thus hold the potential to enhance the therapeutic efficacy of anticancer drugs [16,17,18,19]. The use of external stimuli offers several advantages over endogenous stimuli, as external stimuli are not affected by heterogeneity within the tumor microenvironment and can be applied using noninvasive techniques with excellent spatiotemporal control [20]. Light is an especially desirable trigger that can be remotely applied and accurately spatiotemporally controlled. Moreover, the wavelength, intensity, exposure region and duration of treatment can be easily modulated, thus light has become an increasingly important stimuli for drug delivery systems [21,22,23]. Ultraviolet/visible light has a low depth of penetration in biological tissues, which limits the use of these wavelengths of light for biomedical applications [24,25,26,27]. In contrast, near-infrared (NIR) light in the 600–1000 nm wavelength range can penetrate deeply into tissues, has good photo-stability and does not cause tissue damage, thus NIR has enormous potential as a stimulus for photodynamic therapy [28]. However, in terms of functional criteria, light-controlled nanocarriers for anti-cancer drugs cannot exceed the maximum permissible exposure (MPE) of 0.2 W/cm^2^, 0.317 W/cm^2^, and 0.726 W/cm^2^ after > 10 s laser irradiation at wavelengths of 600–700 nm, 800 nm, and 980 nm, respectively. Light-induced heating above the MPE can lead to thermal pain and cellular damage in the exposed tissues [29,30,31]. 

Combination therapy is more therapeutically effective than individual therapeutic approaches, especially as combination therapy can suppress drug resistance via various mechanisms of action. The combination of chemotherapy with phototherapy, such as photodynamic therapy (PDT), has attracted tremendous attention, due to the potential advantages of extraordinary spatiotemporal controllability, enhanced tumor cell killing ability and reduced systemic toxicity [2,32,33]. PDT is a light-responsive therapeutic approach in which an injected photosensitizer is triggered by precise light irradiation to generate high levels of reactive oxygen species (ROS), which are toxic to tumor cells [34,35,36]. The US Food and Drug Administration (FDA) has approved three photosensitive drugs, photofrin, verteporfin and levulan (5-aminolevulinic acid, 5-ALA), for use in humans. The non-phototoxic prodrug 5-ALA has received the most interest due to its negligible toxicity and rapid renal clearance rate. The drug, 5-ALA, is naturally synthesized in mitochondria as a component of the heme biosynthetic pathway. As a further uptake of 5-ALA by cells, 5-ALA is metabolically converted into the endogenous photosensitizer protoporphyrin IX (PpIX) in the cellular environment [36,37,38]. However, 5-ALA is hydrophilic and does not specifically target tumor cells, thus—undesirably—the prodrug may accumulate in normal tissues and leak into the blood circulation [37]. Various nanocarriers have been used to overcome these issues and boost the tumor-targeting ability and cellular absorption of 5-ALA. For example, Ding and coworkers studied the effect of 5-ALA-induced ROS generation on cellular growth/apoptosis [39]. The nanocarrier reached the tumor site and—following laser irradiation of the tumor—high levels of ROS were produced by conversion of 5-ALA to PpIX and the anticancer drug was simultaneously released from the nanocarrier. Thus, the photodynamic effect induced accumulation of ROS, which directly killed the tumor cells. Ma et al. [40] showed that folic acid-functionalized hollow mesoporous silica nanoparticles could enter cancer cells, release 5-ALA and lead to rapid accumulation of the photosensitizer PpIX for PDT. Thus, polymeric nanoparticles are considered promising vehicles for the delivery of anticancer drugs, including photosensitizers [39,41].

We previously established a series of SRPMs based on adenine-functionalized supramolecular micelles (A-PPG) containing nucleobase motifs and the difunctional oligomer polypropylene glycol (PPG) via a simple one-step chemical reaction. These carriers offer multiple advantages over conventional drug delivery systems, such as the ability to self-assemble into micelles in aqueous solution, extremely stable structures in serum rich-media, and well-controlled intracellular drug release and drug loading capacities due to the pH/temperature-sensitive hydrogen-bonded adenine moieties in the interior of the micelles [42,43,44,45]. Intriguingly, we discovered that these adenine-functionalized A-PPG micelles were selectively internalized into cancer cells in vitro, quickly released the encapsulated drug and significantly reduced the viability of the cancer cells. However, the same effects were not observed in healthy cells, as premature drug release was limited under normal physiological conditions [46,47]. Due to the existence of the self-complementary hydrogen-bonded adenine interactions, the drug-loaded micelles retained stable spherical micellar structures that restricted drug release. At 37 °C, 19% of the drug was released in the first 24 h, 23% after 48 h, with cumulative release of only 25% after 96 h [46]. Consequently, cumulative drug release from A-PPG micelles under normal physiological conditions would be unsatisfactory for drug delivery applications. Photosensitizers lead to the generation of high levels of localized intracellular ROS when activated by laser irradiation. Thus, we hypothesized that generation of ROS by an irradiated photosensitizer would enhance drug release from A-PPG micelles and provide a suitable strategy for synergistic chemo-photodynamic therapy.

**Scheme 1:**
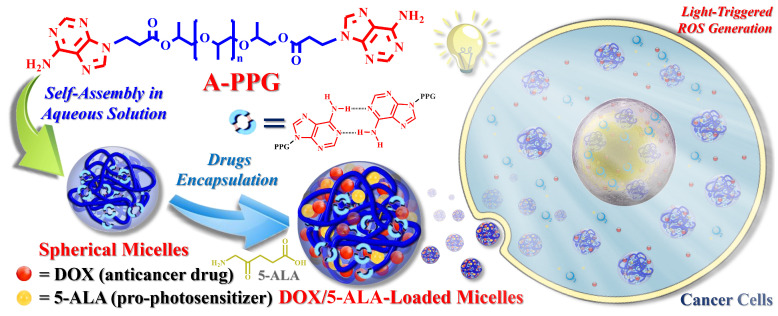
Schematic illustration of light-triggered release of therapeutic agents from adenine-functionalized supramolecular micelles (A-PPG) micelles. Micelles encapsulating doxorubicin (DOX) and the pro-photosensitizer 5-aminolevulinic acid (5-ALA) are internalized and distributed in cancer cells via endocytosis. Activation of the DOX/5-ALA complexes within the micelles with visible light induces degradation of the micelle structure and release of DOX, which results in tumor cell death.

With these objectives in mind, we encapsulated both the chemotherapeutic drug DOX and pro-photosensitizer 5-ALA within A-PPG adenine-functionalized supramolecular micelles. This unique delivery system employs the formation of photosensitive DOX/5-ALA complexes to trigger DOX release from A-PPG micelles upon laser irradiation of the target site via the EPR effect (Scheme 1). DOX/5-ALA-loaded micelles demonstrated high drug-loading capacity and structural stability in serum-containing media due to the hydrogen-bonded adenine–adenine interactions within the micelles. More significantly, in vitro drug release studies demonstrated that laser irradiation of tumor cells triggered rapid, well-controlled drug release from the DOX/5-ALA-loaded micelles. In addition, fluorescence imaging and flow cytometric analysis confirmed that the drug-loaded micelles exhibited significantly improved uptake/intracellular accumulation in tumor cells after laser irradiation, which consequently enhanced the chemotherapeutic efficacy of the micelles in vitro. Thus, formation of the photosensitive DOX/5-ALA complexes within the A-PPG supramolecular micelles enabled light-triggered release of DOX. In response to laser irradiation, light-induced production of ROS in nearby cellular membranes enabled highly selective cellular internalization of the drug DOX and enhanced the efficacy of chemotherapy [46,47]. Thus, A-PPG micelles could serve as a novel anticancer delivery system to effectively inhibit cancer growth and metastasis through the synergistic effect of chemo-photodynamic therapy.

## 2. Results and Discussion

**Figure 1:**
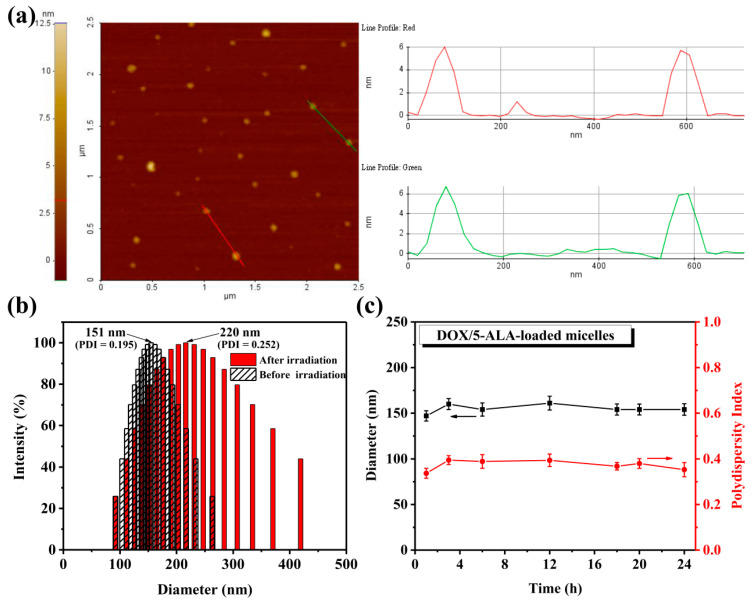
(**a**) Atomic force microscopy (AFM) image of DOX/5-ALA-loaded A-PPG micelles coating on silicon wafer. Cross-section analysis presenting the height distribution along the red and green lines. (**b**) dynamic laser scattering (DLS) analysis of the size distribution of micelles before and after laser irradiation. (**c**) Kinetic stability of DOX/5-ALA-loaded A-PPG micelles in PBS containing 10% fetal bovine serum (FBS) over time at pH 7.4.

A new low-molecular-weight supramolecular polymer micelle, adenine end-capped difunctional oligomeric A-PPG, was successfully prepared using our previously described simple, one-step synthesis [45,46,48]. To achieve a photodynamic chemotherapeutic effect, we selected 5-ALA as a photosensitizer moiety to enhance release of the chemotherapy drug DOX from the micelles at tumor sites, as presented in Scheme 1. The drug and photosensitizer were encapsulated using a dialysis method and UV-Vis spectroscopy at absorbance intensities of 483 nm (DOX) and 420 nm (5-ALA) were used to determine the drug-loading content (DLC) and drug-loading efficiency (DLE). The DLC values of DOX and 5-ALA gradually increased with the feeding amount of the drugs. As shown in Table S1, the DLC of DOX/5-ALA-loaded micelles as double cargo reached maximum values of 7.11 ± 2.38% for DOX and 6.06 ± 2.01% for 5-ALA. The DLC and particle diameters of single-cargo DOX-loaded A-PPG micelles are discussed in our previous study [47]. The hydrodynamic size and morphology of double-cargo DOX/5-ALA-loaded A-PPG micelles were determined by dynamic laser scattering (DLS) and atomic force microscopy (AFM) to optimize the photo-regulated micelles. As illustrated in Figure 1a,b, AFM and DLS revealed DOX/5-ALA-loaded A-PPG micelles had a spherical morphology prior to laser irradiation. In addition, the average particle size of DOX/5-ALA-loaded A-PPG micelles was 151 nm (polydispersity index (PDI) = 0.195). Upon laser irradiation for 120 s (light source: 60 mW/cm^2^), the particle size immediately increased and reached 220 nm (PDI = 0.252; Figure 1b), which implies that structural relaxation of the DOX/5-ALA complexes within the micelles by irradiation with visible light may induce micellar destabilization [49,50], thus resulting in rapid release of DOX from the micelles accompanied with significant increment of the average particle size and distribution. These observations were also consistent with the ultraviolet-visible (UV-Vis) and photoluminescence (PL) results (Figure S1; see Supplementary Materials for details). Taken together, these results clearly demonstrate that the structure of the DOX/5-ALA-loaded A-PPG micelles was disrupted by laser irradiation-induced dissociation of noncovalent DOX/5-ALA complexes [49,50]. Thus, we assessed the stability of DOX/5-ALA-loaded A-PPG micelles in aqueous physiological environments. DOX/5-ALA-loaded A-PPG micelles were added to PBS containing 10 *v%* fetal bovine serum (FBS), which functions as an effective nanoparticle-destabilizing agent, and subjected to DLS at 25 °C [15,51]. As shown in Figure 1c, the particle size distribution of DOX/5-ALA-loaded A-PPG micelles remained almost unchanged after 24 h exposure to FBS, suggesting the extremely stable self-complementary hydrogen-bonding interactions between the adenine moieties of the micelles strongly encapsulate DOX under normal physiological conditions.

**Figure 2:**
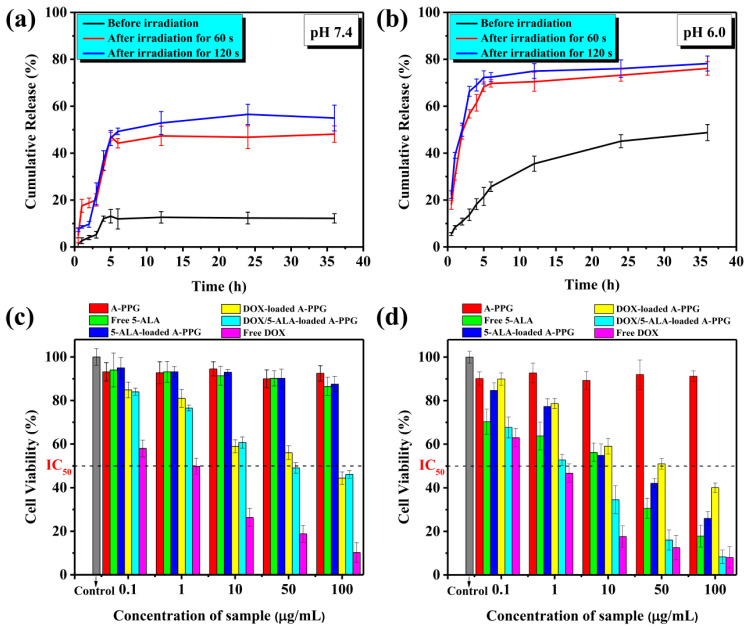
Cumulative DOX release profiles of DOX/5-ALA-loaded A-PPG micelles in PBS before and after irradiation for various periods of time (635 nm light, 60 mW/cm^2^) at (**a**) pH 7.4 and (**b**) pH 6.0. In vitro cell viability of HeLa cells at 24 h after incubation with A-PPG micelles, DOX, 5-ALA, and single-cargo or double-cargo DOX/5-ALA-loaded A-PPG micelles (**c**) before irradiation and (**d**) after irradiation.

In our previous study, we observed low cumulative drug release (less than 28% after 96 h) from drug-loaded A-PPG micelles under physiological conditions [46]. Thus, we investigated the ability of light irradiation to induce release of DOX from DOX/5-ALA-loaded A-PPG micelles in PBS to further explore the release behavior of the system in vitro. The cumulative release profiles conclusively demonstrated that the DOX release profile is closely related to the duration of irradiation and the presence of an acidic environment as a co-effect [15,23]. For example, under normal physiological conditions (pH 7.4), 48% and 54% of the total DOX was released after 60 s and 120 s light irradiation, respectively. In contrast, only 12% of DOX was released from non-irradiated micelles after 36 h (Figure 2a). These data indicate that light irradiation triggered drug release under normal physiological conditions. Moreover, more rapid DOX release was observed when the environmental pH was decreased to 6.5 or 6.0 to mimic the acidic tumor microenvironment: after 60 s and 120 s irradiation, burst release of DOX after 4 h reached 34% and 48% at pH 6.5 and 61% and 69% at pH 6.0, with sustained, continuous release of 66% and 72% (pH 6.5) and 76% and 78% (pH 6.0) achieved by 36 h, respectively—compared to 39% and 48% for non-irradiated micelles at pH 6.5 and pH 6.0 within 36 h, respectively (Figure 2b and Figure S2). These results indicate that light irradiation more rapidly destabilized the structure of the micelles and facilitated DOX release in the acidic pH microenvironment of the cancer cells, demonstrating the existence of a co-dependent effect between light irradiation and the acidic environment. These results further indicate that the presence of the DOX/5-ALA complexes within the micellar structures is the most important factor related to the achievement of light-triggered drug-release properties (Figure S1). Overall, these findings confirm our hypothesis that the combination of light-triggered external stimulus with an acidic pH as an internal stimulus could enhance drug release in cancer tissues and reduce the adverse effects in normal tissues [47,49,50].

To evaluate the combined therapeutic effect of 5-ALA-based PDT and DOX-mediated chemotherapy, HeLa cells were incubated with various micelles and analyzed using the methyl thiazolyl-tetrazolium (MTT) assay. As indicated in Figure 2c,d, A-PPG micelles exerted negligible toxicity towards HeLa cells over the entire concentration range tested, suggesting good biocompatibility. Similarly, free 5-ALA and non-irradiated single-cargo 5-ALA-loaded micelles led to negligible toxicity; 5-ALA only inhibits cell proliferation in a dose-dependent manner upon light irradiation, consistent with the research conducted by Tong et al. [52] However, in the absence of irradiation, single-cargo DOX-loaded A-PPG micelles and double-cargo DOX/5-ALA-loaded A-PPG micelles had half maximal inhibitory concentration (IC_50_) values of 74.8 ± 3.1 μg/mL and 50.1 ± 2.3 μg/mL, respectively, in HeLa cells (Figure 2c). These results imply that, under normal conditions, the chemotherapy agent DOX was slowly released from the loaded micelles in the cancer cell environment.

Surprisingly, double-cargo DOX/5-ALA-loaded A-PPG micelles rapidly released DOX after irradiation and led to higher cytotoxicity than non-irradiated micelles containing equivalent concentrations of the drug. Regardless of light irradiation, the double-cargo DOX/5-ALA-loaded A-PPG micelles more obviously inhibited the growth of HeLa cells (Figure 2d). However, irradiation with light dramatically reduced the IC_50_ of DOX/5-ALA-loaded A-PPG micelles to 2.0 ± 2.8 μg/mL, single-cargo DOX-loaded A-PPG micelles to 72.3 ± 3.2 μg/mL and single-cargo 5-ALA-loaded A-PPG micelles to 55.5 ± 2.4 μg/mL. The irradiated double-cargo DOX/5-ALA-loaded A-PPG micelles were more cytotoxic towards HeLa cells than the same concentrations of single-cargo DOX-loaded or 5-ALA-loaded A-PPG micelles, indicating irradiation-induced conversion of 5-ALA to PpIX led to intracellular production of high levels of ROS, which destroyed the structure of the micelles and led to rapid release of DOX [5,44]. In contrast, HeLa cells treated with free DOX and 5-ALA demonstrated IC_50_ values of 0.93 ± 3.04 μg/mL and 18.95 ± 6.3 μg/mL (Figure 2d). These results imply that the slightly lower cytotoxicity of double-cargo DOX/5-ALA-loaded A-PPG micelles compared to free DOX may be attributed to delayed internalization of the micelle-loaded drug and the time required for DOX to be released from A-PPG micelles into the cells [41]. Overall, these results demonstrate that double-cargo DOX/5-ALA-loaded A-PPG micelles efficiently release encapsulated DOX in response to light irradiation-induced generation of ROS, demonstrating external laser irradiation induced a synergistic chemo-photodynamic phototoxic effect.

**Figure 3:**
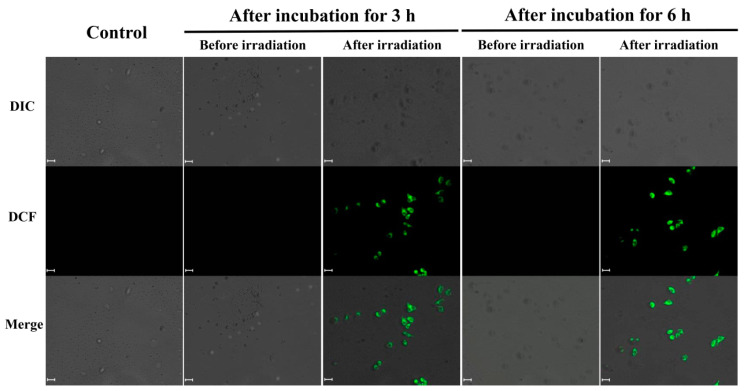
Singlet oxygen production detected by fluorescence of DCF in HeLa cells exposed to DOX/5-ALA-loaded A-PPG micelles before and after irradiation with 635 nm light (60 mW/cm^2^). The differential interference contrast (DIC) channel, 2,7-dichlorofluorescein (DCF) channel and merged images are presented from left to right. All scale bars represent 20 μm.

As irradiation-induced generation of ROS by a photosensitizer is crucial to PDT, 2,7-dichlorofluorescein diacetate (DCFH-DA) was employed as a marker to assess the production of ROS using confocal laser scanning microscopy (CLSM). In living cells, DCFH-DA is converted to DCFH, which is oxidized to fluorescent (green) 2,7-dichlorofluorescein (DCF) in the presence of ROS [42,53]. Confirmation of ROS generation is a crucial element of assessing the effectiveness of PDT. As shown in Figure 3, non-irradiated HeLa cells incubated with DOX/5-ALA-loaded A-PPG micelles (1.5 μg/mL) for 3 h and 6 h did not exhibit green DCF fluorescent staining. In contrast, cells incubated with the same micelles that were irradiated for 120 s exhibited intense green DCF fluorescence. Phua et al. [20] described how a prodrug-based supramolecular nanosystem led to the appearance of green fluorescence in cells after 4 h incubation with photosensitizer-loaded nanoparticles. In contrast, our system led to slightly more rapid appearance of green fluorescence, after 3 h. These results suggest that DOX/5-ALA-loaded A-PPG led to rapid generation of ROS within the light-irradiated cells, due to the synergistic ability of external light stimuli to generate ROS and the acidic intracellular environment of cancer cells to dissociate the hydrogen-bonded adenine dimers and disrupt the micellar structures.

**Figure 4:**
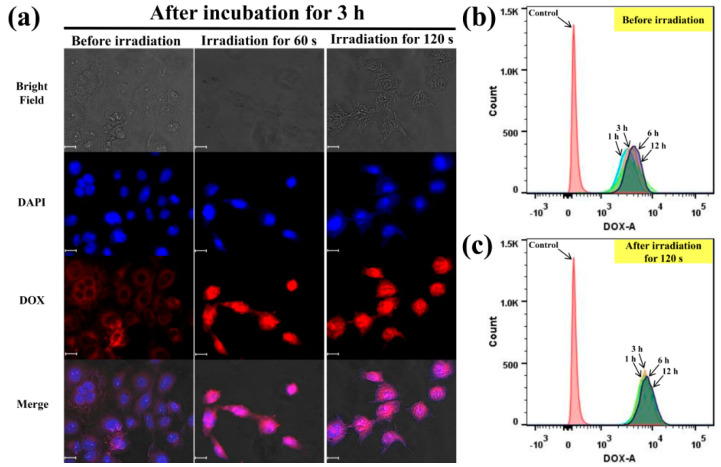
CLSM images of HeLa cells incubated with DOX/5-ALA-loaded A-PPG micelles at 3 h after irradiation. HeLa cells were stained with the nuclear marker DAPI (blue); conjugated DOX emits red fluorescence (λex = 480 nm). All scale bars in (**a**) represent 20 μm. Flow cytometric profiles of HeLa cells incubated with DOX/5-ALA-loaded A-PPG micelles and then (**b**) not irradiated or (**c**) irradiated for various periods of time.

To obtain further insight into the synergistic photo-chemotherapeutic effect of DOX/5-ALA-loaded A-PPG micelles, we assessed the cellular uptake of the micelles using CLSM and flow cytometry. As indicated in Figure 4a and Figure S3a, in HeLa cells incubated with DOX/5-ALA-loaded A-PPG micelles (1.5 μg/mL) for 3 h and 6 h that were not exposed to light irradiation, the micelles were only localized in the cytoplasm of the cancer cells. These results indicate the DOX/5-ALA-loaded A-PPG micelles were internalized into the cancer cells before irradiation, which delayed the entry of DOX into the nucleus. Importantly, after irradiation of the cell cultures for 60 s or 120 s, strong DOX fluorescence was rapidly detected inside the nuclei of the tumor cells incubated with DOX/5-ALA-loaded A-PPG micelles at 3 h and 6 h after irradiation. These results suggest that laser irradiation rapidly destabilized the structure of the micelles by activating PpIX-mediated production of ROS, which in turn led to the rapid release and accumulation of DOX in the nuclei of the cancer cells.

We conducted a flow cytometry analysis to further quantify cellular uptake. As shown in Figure 4b,c, flow cytometry confirmed the results of the CLSM and intracellular ROS assays, as 120 s laser irradiation improved cellular uptake of the double-cargo A-PPG micelles compared to non-irradiated cell cultures. Finally, we asked whether activation of ROS by laser irradiation affected the cellular uptake of double-cargo DOX/5-ALA-loaded A-PPG micelles by plotting the average fluorescence intensity of DOX versus incubation time. Up to 12 h, the average cellular uptake intensity of double-cargo DOX/5-ALA-loaded A-PPG micelles in HeLa cells was approximately two-fold higher after laser irradiation than in non-irradiated cells (Figure S3b). This further implies that DOX/5-ALA-loaded A-PPG micelles were effectively taken up into HeLa cells, possibly due to light irradiation-induced activation of ROS generation by conversion of 5-ALA to PpIX, which disrupted the micellar structures and led to rapid release of DOX into the cancer cells. Notably, these findings further confirm our previous observations that supramolecular A-PPG micelles can be selectively internalized by HeLa cells to efficiently reduce the viability of cancer cells, while exerting minimal cytotoxicity in normal cells [46,47]. Thus, the combination of light-triggered and selective chemotherapy within the A-PPG system not only significantly improves controlled drug delivery and release, but may also enhance the efficacy of chemotherapy.

**Figure 5:**
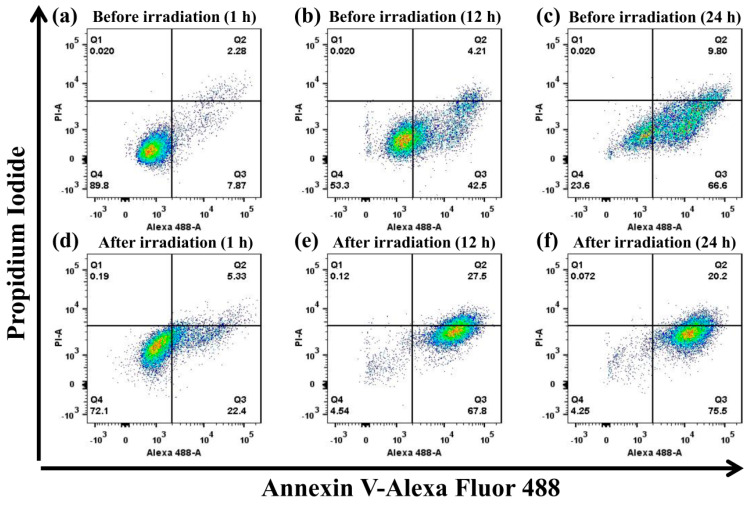
Flow cytometric analysis of the mechanisms of cell death induced by DOX/5-ALA-loaded A-PPG micelles in HeLa cells at 1, 12 and 24 h before irradiation (**a**–**c**) and after irradiation for 120 s (**d–f**). The numbers in the profiles indicate the percentages of cells in each quadrant.

To further investigate the endocytic pathways and mechanisms involved in the therapeutic efficacy of laser-irradiated dual-cargo-loaded A-PPG micelles, we a performed flow cytometric analysis of HeLa cells incubated with DOX/5-ALA-loaded micelles for 1, 12 or 24 h before and after irradiation for 120 s. PI and Annexin V-Alexa Flour 488 double staining were used to detect and quantify apoptotic cells and necrotic cells [14,54]. As shown in Figure 5a–c, when HeLa cells were incubated with DOX/5-ALA-loaded A-PPG micelles for 24 h, only 66.6% of cells were early apoptotic and 9.80% were late apoptotic, suggesting that non-irradiated DOX/5-ALA-loaded A-PPG micelles were not rapidly transported into the HeLa cells and only induced low levels of early/late apoptosis. Encouragingly, after 120 s irradiation, higher proportions of early apoptotic (75.5%) and late apoptotic (20.2%) cells were observed at 24 h in HeLa cells incubated with DOX/5-ALA-loaded A-PPG micelles (Figure 5d–f). These results suggest laser irradiation, as an external stimulus, induced rapid release of DOX from DOX/5-ALA-loaded A-PPG micelles and effectively promoted apoptotic cell death, consistent with the results of the MTT assay. In addition, these experiments demonstrated conversion of ALA to PpIX does not induce apoptosis in the absence of light irradiation [5]. Overall, this study demonstrates that visible light-responsive A-PPG micelles can effectively control the delivery and release of drugs at tumor sites and specifically enhance the cytotoxicity of anticancer drugs in tumor cells [46,47], and thus may represent an attractive system for targeted drug delivery applications in cancer therapy.

## 3. Materials and Methods

### 3.1. Materials

Poly(propylene glycol) diacrylate (average molecular weight, ~800 g/mol), adenine (≥99.5% purity), potassium tert-butoxide (t-BuOK), dimethylformamide (DMF) and all other chemicals and reagents were purchased from Sigma-Aldrich (St. Louis, MO, USA) at the highest purity available. Material syntheses and characterization were performed as previously described in detail [43,46].

### 3.2. Characterization

The average particle size and zeta potentials of samples were assessed using a Nano Brook 90 Plus PALS (Brookhaven Instruments Corp., Holtsville, NY, USA). DLS measurements were carried out at 25 °C; samples were incubated at 25 °C for at least 20–30 min before measurement. AFM was used to assess the morphology of DOX-loaded micelles. Diluted samples of non-irradiated DOX/5-ALA-loaded micelles and irradiated DOX/5-ALA-loaded micelles (after at least 30 min at room temperature) in deionized water were spin-coated onto silicon wafers (125 nm), dried in a vacuum oven at 25 °C, and AFM images were captured using a tapping-mode AFM (NX10; AFM Park Systems, Suwon, South Korea). The V-730 ultraviolet-visible (UV-Vis) spectrophotometers (Jasco, Tokyo, Japan) and F4500 photoluminescence (PL) spectrometer (Hitachi, Tokyo, Japan) were used to evaluate the optical properties of aqueous micellar solutions. Photodynamic analysis was conducted with a PROVA 500 (OPAS, Taiwan) using the following settings: light source (fiber coupled laser system; LaserLab, Taiwan) at 7.2 J/cm^−2^, 635 nm; irradiation intensity, 60 mW/cm^2^; sample distance, 12 cm from UV lamp (sample site, length × width: 15 cm × 15 cm).

### 3.3. Preparation of DOX/5-ALA-Loaded A-PPG Micelles

DOX and 5-ALA were directly encapsulated into the polymeric micelles via a simple dialysis method. Varied amounts of doxorubicin hydrochloride (DOX.HCL) and/or 5-ALA and the polymer micelles were mixed (ranging from 0.25 to 1.5 mg), dissolved in DMF, then 0.2 mL of trimethylamine (TEA) was added and stirred for 24 h. The mixtures were dialyzed against PBS (pH 7.4, 10 mM) in a dialysis membrane with a molecular weight cut off (MWCO) of 1000 Da for 24 h at 15 °C to remove unloaded free DOX, 5-ALA and organic solvent.

The amount of DOX loaded into the polymeric micelles was directly determined by UV-Vis spectroscopy at λ = 483 nm (the absorbance intensity of DOX). Briefly, 2 mg of lyophilized DOX-loaded micelles were dissolved in PBS to determine the drug loading content (DLC) and loading efficiency (DLE).

The 5-ALA content of the micelles was determined using 2,4,6-trinitrobenzene sulfonic acid (TNBS) [2,52,55]. Briefly, 25 μL of 0.03 M TNBS was added to 1 mL samples in borate buffer and analyzed by UV-Vis spectroscopy at λ = 420 nm (the absorbance intensity of 5-ALA; the 420 nm wavelength region was removed from the TNBS absorption spectra). A gradient series of 5-ALA standard solutions was used to prepare the calibration curve.

The drug-loading content (DLC) and drug-loading efficiency (DLE) were calculated using:

(1) $$(\mathrm{DLC})\;\%\;=\;\frac{\mathrm{Weight}\;\mathrm{of}\;\mathrm{drug}\;\mathrm{loaded}\;\mathrm{in}\;\mathrm{polymeric}\;\mathrm{micelles}}{\mathrm{Weight}\;\mathrm{of}\;\mathrm{drug}\;\mathrm{loaded}\;\mathrm{polymeric}\;\mathrm{micelles}\;}\;\times \;100$$

(2) $$(\mathrm{DLE})\;\%\;=\;\frac{\mathrm{Weight}\;\mathrm{of}\;\mathrm{drug}\;\mathrm{loaded}\;\mathrm{in}\;\mathrm{polymeric}\;\mathrm{micelles}\;}{\mathrm{weight}\;\mathrm{of}\;\mathrm{drug}\;\mathrm{input}\;}\;\times \;100$$

### 3.4. Evaluation of the Stability of DOX/5-ALA-Loaded Micelles

Fetal bovine serum (FBS) is commonly used as a biological micelle-destabilizing agent to assess the kinetic stability of micelles in aqueous environments. Briefly, DOX/5-ALA-loaded micelles were mixed with DMEM (pH 7.4, 10 mM) or DMEM containing 10% fetal bovine serum. The changes in the hydrodynamic diameter and PDI of the micelles were monitored over 24 h at 25 °C by DLS. All results are expressed as the mean and standard deviation (± SD) of three independent experiments with three replicates each.

### 3.5. In Vitro DOX Release Assay

In vitro drug release was determined using a dialysis method in PBS at pH 7.4, 6.5 or 6.0 at 37 °C. Briefly, DOX/5-ALA-loaded micelle solutions were dialyzed in 1000-Da MWCO dialysis membrane with stirring at 100 rpm at 37 °C for 36 h. Samples were removed at predetermined sampling times before irradiation or 60 s or 120 s after irradiation at 635 nm (light source: 60 mW/cm^2^) and replaced with fresh PBS. The cumulative amount of DOX released was measured by UV-Vis spectroscopy at 486 nm, compared to a standard calibration curve for free DOX in PBS and plotted as a function of time, using:

(3) $$\mathrm{Cumulative}\;\mathrm{drug}\;\mathrm{release}\;\%\;=\;\frac{Wt\times 100}{W}$$

where *Wt* is the amount of DOX released at time *t* and *W* is the total DOX loaded into A-PPG micelles. The results are presented the mean ± SD of three independent experiments.

### 3.6. Cell Culture

HeLa cells were cultured in Dulbecco’s modified Eagle’s medium (DMEM) supplemented with 10% FBS (Mediatech, Fairfax, VA, USA) and 1% penicillin-streptomycin (Invitrogen, Carlsbad, CA, USA) in a humidified atmosphere containing 5% CO_2_ at 37 °C.

### 3.7. In Vitro Cytotoxicity Studies

Briefly, HeLa cells were seeded into 96-well plates at 1 × 10^6^ cells per well in 100 μL DMEM. After 24 h, the media was removed and 100 μL DMEM containing various concentrations of DOX (0.01 to 100 μg/mL) was added, incubated for 4 h, and the culture plates were irradiated at 635 nm for 120 s (light source 60 mW/cm^2^) or not irradiated. The cells were cultured for 18 h, 20 μL of MTT solution in PBS (5 mg/mL) was added to each well, incubated for 4 h, 150 μL DMSO was added per well to dissolve the MTT-generated formazan, and the absorbance values were determined using an ELISA microplate reader (Thermo Fisher Scientific, Waltham, MA, USA) at 570 nm. All results are reported as the mean ± SD of three independent experiments with three replicates each. IC_50_ values were determined by plotting cell viability (%) versus DOX concentration (μg/mL).

### 3.8. Intracellular ROS Assay

Intracellular ROS generation in laser-irradiated cells was detected using dichlorofluorescein diacetate (DCFH-DA) [20,52,56]. Briefly, HeLa cells were seeded into 6-well plates at a density of 2 × 10^5^ cells per well in 2.0 mL of complete DMEM containing 10% FBS and 1% penicillin-streptomycin (Invitrogen). After 24 h, the culture media was removed and the cells were incubated with empty micelles or DOX/5-ALA-loaded micelles in complete DMEM for 3 or 6 h, washed with PBS, and DMEM supplemented with DCFH-DA (final concentration: 1 × 10^−5^ M) was added to the wells. Thirty minutes later, the cells were thoroughly washed with PBS to remove residual DCFH-DA and irradiated for 120 s (light source 60 mW/cm^2^) or not irradiated. Cells were imaged by confocal microscopy (iRiS™ Digital Cell Imaging System, Logos Bio Systems, Gyeonggi-do, South Korea).

### 3.9. Analysis of Cellular Uptake by Confocal Laser Scanning Microscopy

HeLa cells were seeded into 6-well plates at approximately 1 × 10^6^ cells per well in 2 mL complete DMEM and cultured for 24 h. Then, the media was replaced with 2 mL fresh media containing DOX/5-ALA-loaded micelles, and the cells were irradiated for 60 s or 120 s (light source 60 mW/cm^2^) or not irradiated, incubated for 3 or 6 h, the medium was removed, cells were washed in PBS, fixed in 4% formaldehyde for 15 min, washed with PBS (pH 7.4), stained with 4’,6-diamidino-2-phenylindole (DAPI; Sigma-Aldrich) for 30 min to visualize nuclei, and the cells were examined by confocal laser scanning microscopy (iRiS™ Digital Cell Imaging System).

### 3.10. Detection of DOX Fluorescence Intensity by Flow Cytometry

HeLa cells were seeded into 6-well plates at 1 × 10^6^ per well in 2 mL complete DMEM media, cultured for 24 h, then irradiated for 120 s (light source 60 mW/cm^2^) or not irradiated, incubated for 1, 3, 6 or 12 h, then the culture medium was removed and the cells were washed three times with PBS and detached with trypsin. Then, 2.0 mL of PBS was added to each well, the cell suspensions were centrifuged for 4 min at 3000 rpm, the supernatants were removed, and the cells were re-suspended in cold PBS and analyzed by flow cytometry (FACSAriaTM III; BD Biosciences, San Jose, CA, USA).

### 3.11. Flow Cytometric Analysis of Apoptosis by Annexin V/PI Double Staining

HeLa cells were seeded into 6-well plates at 1 × 10^6^ cells per well in DMEM, cultured for 24 h, irradiated for 120 s (light source 60 mW/cm^2^) or not irradiated, cultured for 1, 12 or 24 h, washed, re-suspended in PBS, harvested, washed twice with cold PBS, stained using Annexin V/PI for 15 min at 37 °C in the dark, and examined by flow cytometry (FACSAriaTM III).

### 3.12. Statistical Analysis

All experiments were repeated independently three times. Data are presented as the mean ± SD of three independent experiments.

## 4. Conclusions

We demonstrate that double-cargo DOX and 5-ALA-loaded adenine-functionalized supramolecular micelles combined with chemotherapy and PDT achieved the precise, light-triggered, spatiotemporally controlled release of anticancer drugs. Since the polymer structure contains self-complementary hydrogen-bonding adenine groups, A-PPG micelles can self-assemble in aqueous media and effectively encapsulate anticancer drugs with extremely long-term stability in serum-containing media. Laser irradiation of cells incubated with the micelles triggered conversion of 5-ALA to the photosensitizer PpIX and led to the generation of high levels of ROS, which destabilized the micelles and promoted the rapid release of DOX. In vitro assays confirmed that laser irradiation significantly increased the cytotoxicity of double-cargo DOX/5-ALA-loaded A-PPG micelles compared to non-irradiated cells incubated with the micelles. Importantly, flow cytometric analysis confirmed that the light-irradiated double-cargo A-PPG micelles were rapidly endocytosed by cells, which therefore accelerated apoptotic cell death. Thus, this promising strategy could potentially be utilized to establish a clinically effective combined chemo-photodynamic therapy for cancer.

## Data Availability

The data that supports the findings of this study are available within the article and its supplementary materials.

## Supplementary Materials

The following are available online at https://www.mdpi.com/1422-0067/22/1/154/s1, Figure S1: UV-Vis spectra and PL spectra of DOX-loaded and DOX/5-ALA A-PPG micelles in aqueous solution before and after irradiation, Figure S2: Cumulative DOX release profile of non-irradiated and irradiated DOX/5-ALA-loaded A-PPG micelles in PBS at pH 6.5, Figure S3: CLSM images and flow cytometric mean fluorescence intensity of HeLa cells incubated with double-cargo DOX/5-ALA-loaded A-PPG micelles, Table S1: Particle size, zeta potential, drug-loading content (DLC) and drug-loading efficiency (DLE) of single- and double-cargo-loaded A-PPG micelles.
