# Supplementary material for: Photo-Responsive Supramolecular Micelles for Controlled Drug Release and Improved Chemotherapy
Source: Int J Mol Sci. 2020 Dec 25;22(1):154. doi: 10.3390/ijms22010154 (PMC7795671; doi:10.3390/ijms22010154)
Supplement: Supplementary file 1 [file ijms-22-00154-s001.pdf]

Supporting Information

Photo-Responsive Supramolecular Micelles for Controlled Drug Release and Improved Chemotherapy

Fasih Bintang Ilhami,[a,b] Kai-Chen Peng,[b] Yi-Shiuan Chang,[a] Yihalem Abebe Alemayehu,[a] Hsieh-Chih Tsai,[a,c,d] Juin-Yih Lai,[a,c,d] Yu-Hsuan Chiao,[e] Chen-Yu Kao[b]* and Chih-Chia Cheng[a,c]*

a. Graduate Institute of Applied Science and Technology, National Taiwan University of Science and Technology, Taipei 10607, Taiwan.

b. Graduate Institute of Biomedical Engineering, National Taiwan University of Science and Technology, Taipei 10607, Taiwan.

c. Advanced Membrane Materials Research Center, National Taiwan University of Science and Technology, Taipei 10607, Taiwan.

d. R&D Center for Membrane Technology, Chung Yuan Christian University, Chungli, Taoyuan 32043, Taiwan.

e. Department of Chemical Engineering, University of Arkansas, Fayetteville, AR 72701, United States.

* Correspondence: ckao@mail.ntust.edu.tw; cccheng@mail.ntust.edu.tw

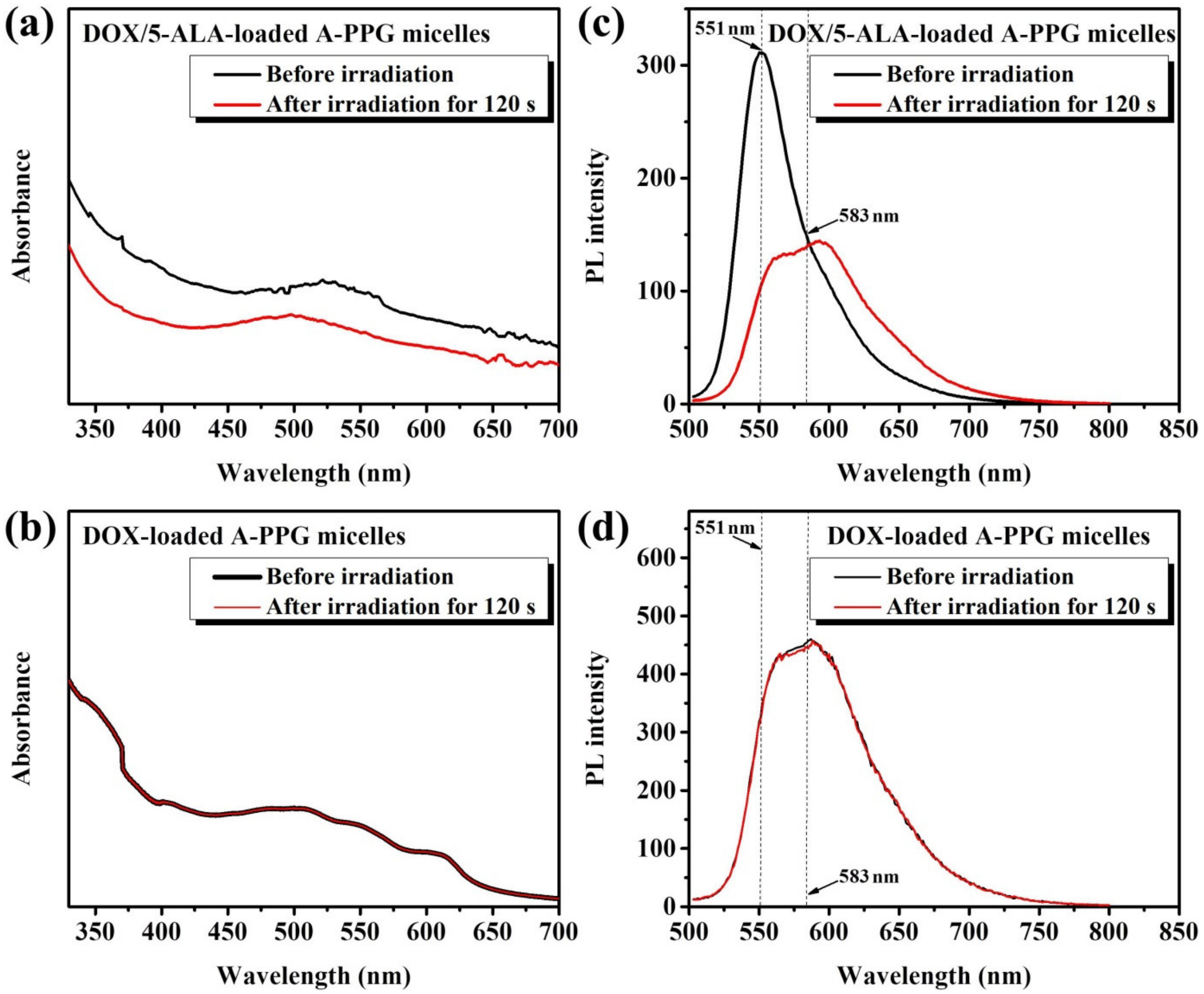

Figure S1: (a, b) UV-Vis spectra and **(c, d)** PL spectra of DOX-loaded and DOX/5-ALA A-PPG micelles in aqueous solution before and after irradiation.

We have performed spectroscopic analysis of DOX/5-ALA-loaded A-PPG micelles before and after laser irradiation in order to understand their effects on light sensitivity. As shown in Figure S1d, single-cargo DOX-loaded A-PPG micelles exhibited a broad emission peak centered around 583 nm, whereas the emission peak of the double-cargo DOX/5-ALA-loaded A-PPG micelles was substantially blue-shifted (to 551 nm) and became sharper and higher intensity (Figure S1c). These results could possibly be attributed to the presence of electron transfer (ET) between DOX and 5-ALAwithin the micellar structure,[55,56] suggesting that the photo-

generated excitons in DOX can transfer to the structural interface of the 5-ALA via the ET process and lead to efficient recombination of photoexcited charge carriers in the 5-ALA, thus resulting in the enhanced emission properties. More surprisingly, double-cargo DOX/5-ALA-loaded A-PPG micelles after irradiation showed significant reduction/alteration in the peak intensity and position of the UV-Vis and PL spectra, respectively (Figure S1a and S1c). In addition, fluorescence spectra reverted back completely to the original form of DOX-loaded A-PPG micelles (Figure S1c and S1d), implying that the ET fluorescence from DOX/5-ALA complexes is completely destroyed by the irradiation, thus the micellar structure rapidly convert into an "unstable" state and then accelerate the release rate of the drug from micellar nanocarriers. By contrast, irradiation process did not affect the absorbance and fluorescence peaks of single-cargo DOX-loaded A-PPG micelles due to the absence of the DOX/5-ALA complexes within the micelles (Figure S1b and S1d), further suggesting that DOX-loaded micelles are insensitive to visible light but react to pH changes in their environment (Figures 2a, 2b and S2).

Based on the above findings, these observations indicate that the ET fluorescence characteristic of the DOX/5-ALA complexes within the micellar structures is the most important factor related to the achievement of light-triggered drug-release properties. Although the exact mechanism of action of dissociated DOX/5-ALA complexes under irradiation is not yet clear, a range of research projects both experimental and theoretical studies are currently underway to investigate the fluorescence behavior,

kinetic characteristic and dynamic simulation of DOX/5-ALA-loaded A-PPG micelles in water before and after irradiation.

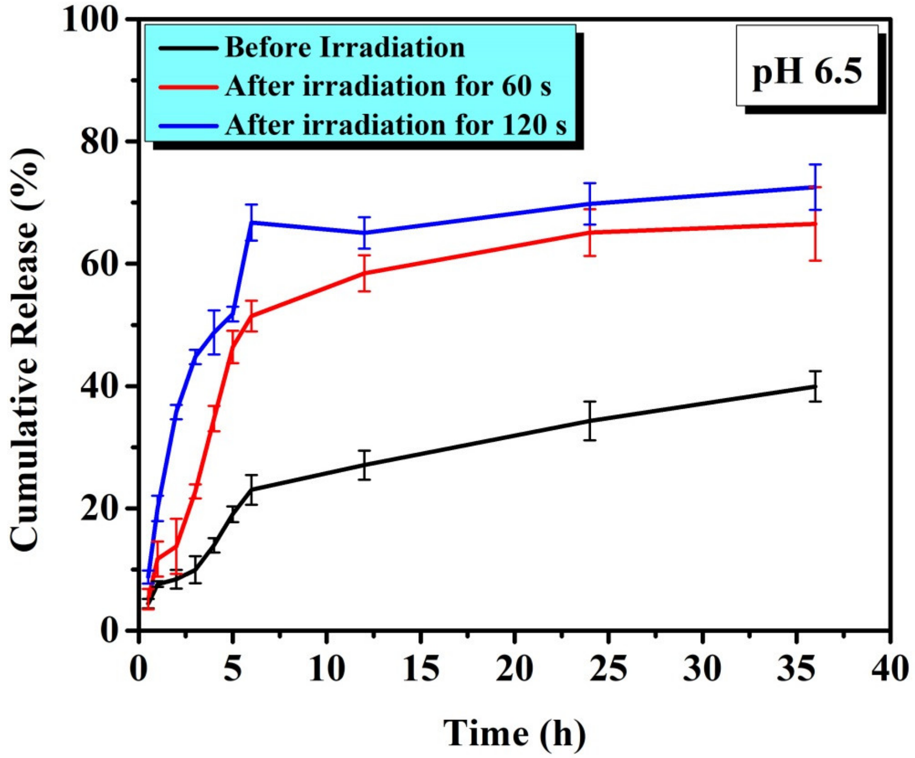

Figure S2: Cumulative DOX release profile of non-irradiated and irradiated DOX/5-ALA-loaded A-PPG micelles in PBS at pH 6.5; micelles were irradiated at 635 nm (60 mw/cm^2) for 60 s or 120 s.

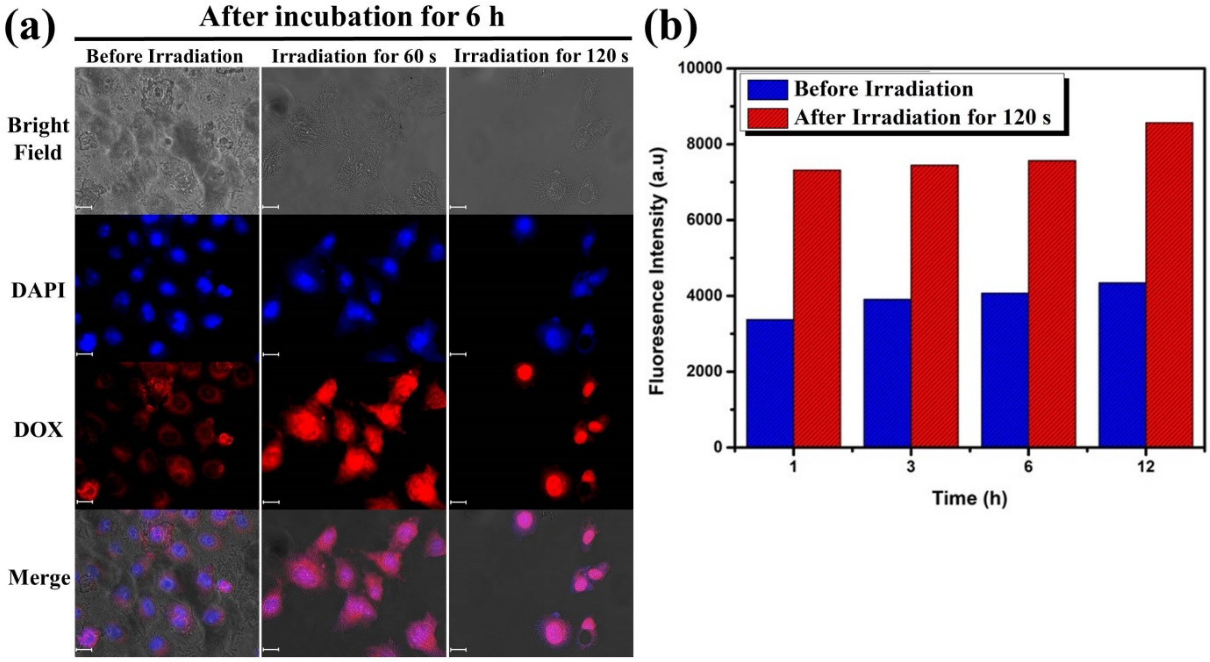

Figure S3: (a) CLSM images of HeLa cells incubated with double-cargo DOX/5-ALA-loaded A-PPG micelles. Cell nuclei were stained with 4′,6-diamidino-2-phenylindole (DAPI, blue fluorescence); red signals indicate the characteristic fluorescence of DOX at 6 h. All scale bars in **(a)** are 20 μm. (**b**) Flow cytometric mean fluorescence intensity of HeLa cells incubated with non-irradiated or irradiated (120 s) DOX/5-ALA-loaded A-PPG micelles for various periods of time (**Figure 4b** and **4c**).

Table S1: Particle size, zeta potential, drug-loading content (DLC) and drug-loading efficiency (DLE) of single- and double-cargo-loaded A-PPG micelles.

DOX/5-ALA-loaded sample[a]	DOX : 5-ALA : A-PPG (weight ratio)	Diameter ± SD (nm)	Zeta potential ± SD (mv)	DOX		5-ALA	
				DLC ± SD (%)[b]	DLE ± SD (%)[c]	DLC ± SD (%)[b]	DLE ± SD (%)[c]
DOX / 5-ALA	1 : 1		-0.11 ± 4.56				
DOX / 5-ALA	1 : 2		4.53 ± 5.87				
DOX / 5-ALA	2 : 1		-2.63 ± 7.07				
A-PPG / 5-ALA	1 : 1	116 ± 2.77	-44.04 ± 7.28			19.3 ± 3.28	16.09 ± 3.77
A-PPG / DOX	1 : 1	121 ± 4.06	25.77 ± 4.48	16.1 ± 4.01	15.4 ± 3.46		
DOX / 5-ALA / A-PPG	0.25 : 0.25 : 3	136 ± 2.18	29.68 ± 5.95	5.37 ± 1.52	21.30 ± 1.22	5.80 ± 5.69	23.12 ± 4.81
DOX / 5-ALA / A-PPG	0.5 : 0.5 : 3	151 ± 2.99	26.62 ± 3.5	5.81 ± 1.03	12.97 ± 1.42	5.88 ± 4.52	13.12 ± 4.09
DOX / 5-ALA / A-PPG	1 : 1 : 3	162 ± 2.94	18.56 ± 3.68	7.11 ± 2.38	9.19 ± 3.36	6.06 ± 2.01	7.74 ± 1.72
DOX / 5-ALA / A-PPG	1.5 : 1.5 : 3	187 ± 2.87	24.43 ± 7.44	3.30 ± 3.71	3.27 ± 4.03	6.76 ± 2.28	6.96 ± 1.46

[a] The micelle concentration in the initial PBS solution was 2 mg/mL. Measurements were performed in PBS (pH 7.4); particle size was measured by DLS.

[b] DLC = drug-loading content.

[c] DLE = drug-loading efficiency
